# Thromboelastometry and organ failure in trauma patients: a prospective cohort study

**DOI:** 10.1186/s13054-014-0687-6

**Published:** 2014-12-25

**Authors:** Marcella CA Müller, Kirsten Balvers, Jan M Binnekade, Nicola Curry, Simon Stanworth, Christine Gaarder, Knut M Kolstadbraaten, Claire Rourke, Karim Brohi, J Carel Goslings, Nicole P Juffermans

**Affiliations:** Department of Intensive Care Medicine, Academic Medical Center, Meibergdreef 9, 1105 AZ Amsterdam, The Netherlands; Department of Surgery, Trauma Unit, Academic Medical Center, Meibergdreef 9, 1105 AZ Amsterdam, The Netherlands; National Health Service Blood and Transplant/Hematology, John Radcliffe Hospital, Headley Way, Oxford, OX3 9BQ UK; Department of Traumatology, Oslo University Hospital, Ullevaal, Nydalen, N-0424 Oslo, Norway; Trauma Sciences, Blizard Institute, Barts and the London School of Medicine and Dentistry, Queen Mary University, Turner Street, London, E1 2AD UK

## Abstract

**Introduction:**

Data on the incidence of a hypercoagulable state in trauma, as measured by thromboelastometry (ROTEM), is limited and the prognostic value of hypercoagulability after trauma on outcome is unclear. We aimed to determine the incidence of hypercoagulability after trauma, and to assess whether early hypercoagulability has prognostic value on the occurrence of multiple organ failure (MOF) and mortality.

**Methods:**

This was a prospective observational cohort study in trauma patients who met the highest trauma level team activation. Hypercoagulability was defined as a *G* value of ≥11.7 dynes/cm^2^ and hypocoagulability as a *G* value of <5.0 dynes/cm^2^. ROTEM was performed on admission and 24 hours later.

**Results:**

A total of 1,010 patients were enrolled and 948 patients were analyzed. Median age was 38 (interquartile range (IQR) 26 to 53), 77% were male and median injury severity score was 13 (IQR 8 to 25). On admission, 7% of the patients were hypercoagulable and 8% were hypocoagulable. Altogether, 10% of patients showed hypercoagulability within the first 24 hours of trauma. Hypocoagulability, but not hypercoagulability, was associated with higher sequential organ failure assessment scores, indicating more severe MOF. Mortality in patients with hypercoagulability was 0%, compared to 7% in normocoagulable and 24% in hypocoagulable patients (*P* <0.001). EXTEM CT, alpha and *G* were predictors for occurrence of MOF and mortality.

**Conclusions:**

The incidence of a hypercoagulable state after trauma is 10% up to 24 hours after admission, which is broadly comparable to the rate of hypocoagulability. Further work in larger studies should define the clinical consequences of identifying hypercoagulability and a possible role for very early, targeted use of anticoagulants.

**Electronic supplementary material:**

The online version of this article (doi:10.1186/s13054-014-0687-6) contains supplementary material, which is available to authorized users.

## Introduction

Major trauma is among the most common causes of death worldwide. Whereas uncontrolled bleeding accounts for 50 to 80% of mortality early following trauma [1,2], multiple organ failure (MOF) is the most important cause of late mortality after trauma [1,3]. Traumatic injury induces a hypocoagulable state, as a result of acute traumatic coagulopathy (ATC) accompanied by loss, consumption and dilution of coagulation factors and fibrinolysis. Hypothermia, shock and acidosis further amplify the derangement of the coagulation system [4]. In addition to reduced hemostatic potential, trauma can also induce a hypercoagulable state [5-7]. Animal experiments have shown that hypercoagulability can arise within hours of the injury [8], a phenomenon confirmed in humans [5,9]. However, uniform definitions of hypercoagulability are lacking and effects of this hypercoagulable state after trauma are not fully elucidated, with studies showing conflicting results. An association with adverse events such as an increased risk of venous thromboembolism has been reported [7,10,11]. However, early hypercoagulability has also been associated with decreased early mortality, which may suggest that hypercoagulability is a functional response in order to reduce blood loss [9].

In sepsis, it has been demonstrated that hypercoagulability, characterized by the formation of microthrombi with concurrent protein C deficiency and impaired fibrinolysis, contributes to MOF and adverse outcome [12,13]. Although sepsis and trauma are different entities, the accompanying coagulopathies show similarities and persistent protein C deficiency after trauma is also associated with occurrence of MOF [14,15]. Shock and hypoperfusion can induce activation of the endothelium and if the patient survives the initial bleeding episode, this can result in a procoagulant state. It is conceivable that therapy of ATC may add to this endogenous response, possibly resulting in an overshoot in coagulation over time, with subsequent enhancement of hypercoagulability and MOF.

Diagnosing hypercoagulability is complex. Thrombin generation tests, or assessment of plasma levels of natural anticoagulants, as protein C, are not readily available for clinical use and not validated to detect hypercoagulability. Thromboelastometry (ROTEM) provides real-time information on all aspects of the coagulation system, including the presence of hypercoagulability [16,17]. The use of thromboelastometry to diagnose hypocoagulability in trauma has frequently been explored in recent years [18-21]. However, reports on the use of ROTEM to detect a hypercoagulable state are scarce.

We aimed to study the incidence of early hypercoagulability in multiple trauma patients and to establish whether hypercoagulability is associated with the occurrence of MOF and mortality. In addition, as transfusion strategies have shifted, we assessed whether transfusion strategy influenced the occurrence of hypercoagulability.

## Methods

### Study design and patients

A prospective observational cohort study was conducted in four level-1 trauma centers in London, Oxford, Oslo and Amsterdam. This study is part of the Activation of Coagulation and Inflammation in Trauma (ACIT) study, an ongoing prospective observational multicenter study in trauma patients. The ethics committees of the Academic Medical Center in Amsterdam, the Netherlands; of the Oslo University Hospital, Oslo, Norway; of the Royal London Hospital, London and of the John Radcliffe Hospital, Oxford, United Kingdom, all reviewed and approved the study. Written informed consent was obtained from all participating patients. All procedures have been performed in accordance with the ethical standards laid down in the 1964 Declaration of Helsinki and its later amendments. Between January 2008 and March 2013, all adult trauma patients (18 years and older) who met the local criteria for highest trauma team level activation were eligible for enrollment in the study. Patients were excluded if arrival at the emergency department (ED) was >2 hours following injury; >2,000 ml of intravenous fluid was administered before ED admission; they were transferred from another hospital or if they had burns covering >5% of total body surface area. Patients were retrospectively excluded if they declined to give consent to use data, were receiving anticoagulation (not including aspirin), or had moderate or severe liver disease or a known bleeding diathesis.

### Data collection

Data were prospectively collected on patient demographics, time from injury to arrival at the ED, mechanism of injury (blunt or penetrating), presence of traumatic brain injury, vital signs on arrival and 24 hours after injury and amount of fluid and blood products within the first 24 hours of injury. Trauma severity was assessed using the injury severity score (ISS) [22]. Furthermore, sequential organ failure assessment (SOFA) scores, with Glasgow coma scale to assess neurologic dysfunction, and mortality rates after 28 days were obtained.

### Thromboelastometry

Thromboelastometric variables were measured with ROTEM (Tem International, Munich, Germany). Citrated blood samples were drawn within 1 hour after arrival in the ED and a second sample was collected 24 hours (±2 hours) after admission. All samples were processed within 1 hour. For EXTEM, 20 μL of 0.2 mol/L CaCl_2_ (star-tem™) and 20 μL of human recombinant tissue factor (*r* EXTEM™) were added to a test vial. Subsequently 300 μL of the citrated blood sample was added. For INTEM, 20 μL of 0.2 mol/L CaCl_2_ (star-tem™) and 20 μL of partial thromboplastin phospholipid made of rabbit brain and ellagic acid (in-tem™) were added as activator to 300 μL of blood in the test cuvette. The electronic pipette program guided all test steps. For both assays, clotting time (CT), clot formation time (CFT), maximum clot firmness (MCF) and alpha angle were recorded. Total clot strength was assessed by *G* as calculated according to the formula: (5,000 × MCF)/100 - MCF and expressed as dynes/cm^2^ [16]. *G* has a curvilinear relation with MCF and reflects the contribution of enzymatic and platelet components to the hemostasis, hereby better reflecting hemostatic potential than individual thromboelastometry parameters [7,23]. *G* has been shown to be valuable in diagnosing hypo- and hypercoagulability [7,16,23]. Hypercoagulability was defined as a *G* value of ≥11.7 dynes/cm^2^ and hypocoagulability as a *G* value of <5.0 dynes/cm^2^ (values provided by manufacturer).

### Outcome variables

Primary outcome was the occurrence of MOF, assessed by the SOFA score, which reliably assesses organ failure in trauma patients [24]. The score awards 0 (normal) to 4 (most abnormal) points for each organ system. MOF was defined by a score of 3 points or more [3]. Secondary outcome was 28-day mortality. In addition, effect of transfusion strategy (ratio of red blood cells (RBC) to fresh frozen plasma (FFP)) on ROTEM profile and occurrence of hypercoagulability was determined.

### Statistics

Continuous normally distributed variables are expressed by their mean and standard deviation. Not normally distributed variables are expressed as medians and their interquartile (IQR) ranges and categorical variables are expressed as n (%). ISS was treated as a continuous variable. Groups are compared by using Student’s *t* test or Mann-Whitney *U* test in case of not normally distributed data.

For comparison of categorical variables, the chi-square test or Fisher’s exact tests are used.

The primary analysis focused on modeling the hypothesized relation between ROTEM-detected hypercoagulability, MOF and mortality in trauma patients. First, univariate logistic regression analysis was used to select independent factors achieving a *P* value ≤0.10, in addition to factors that were deemed clinically important (age, time to ED, presence of traumatic brain injury, injury mechanism, ISS, base excess, systolic blood pressure) in relation to the outcome variables. Subsequently, selected ROTEM factors were entered in a multivariate logistic regression model. Patients who died on admission were not included in the analyses to assess the value of thromboelastometry to predict MOF, while patients who died later were included when a SOFA score was available. All deceased patients were included in the analyses to assess the value of ROTEM to predict mortality.

To compare the effect of transfusion strategies, transfused patients were divided based on RBC:FFP ratio. Statistical significance was considered to be at *P =* 0.05. Analyses were performed using R (version 2 · 3; R Foundation for Statistical Computing, Vienna, Austria). Graphs were created with Prism 5.0 (GraphPad Software, San Diego, CA, USA).

## Results

**Figure 1:**
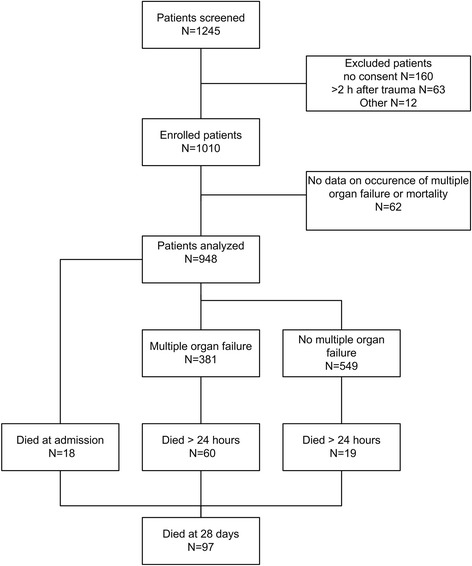
**Flow diagram of inclusion and occurrence of multiple organ failure and mortality.**

During the study period, 1,245 patients were screened and 1,010 patients were enrolled in the study (Figure 1). For 62 of the patients, no data were available on occurrence of MOF or mortality, therefore, analyses were performed in the remaining 948 patients.

Of these, 776 patients were admitted to the hospital (intensive care unit: n = 318 and ward: n = 458) and 76 were discharged home. Patient characteristics are listed in Table 1. The majority of included patients were males experiencing blunt injury. Median age was 38 years and median ISS was 13 (IQR 8 to 25). Eighteen patients died at admission, nine of them had traumatic brain injury (TBI). Of those who died between 24 hours and 28 days, mortality was 21% in TBI and 3.4% in non-TBI patients.

**Table 1:** **Characteristics of patients with hyper-, hypo- and normocoagulable ROTEM profiles at admission**

	**All patients**	**Hypercoagulable** ^**1**^	**Normocoagulable** ^**2**^	**Hypocoagulable** ^**3**^	***P*** **value**
	**N = 886**	**N = 63**	**N = 752**	**N = 71**	
Age (years)	38 [26–53]	44 [33–62]	38 [25–53]	38 [25–54]	<0.05
Sex, male % (n)	78 (688)	60 (38)	80 (599)	72 (51)	<0.001
Time to ED (minutes)	71 [53–90]	71 [46–86]	70 [53–88]	80 [60–100]	0.05
Trauma mechanism, blunt % (n)	81 (722)	81 (51)	82 (619)	86 (61)	0.69
Brain injury, % (n)	26 (233)	23 (14)	27 (193)	38 (26)	0.09
ISS	13 [6–25]	9 [5–17]	13 [5–25]	20 [10–39]	<0.001
Systolic BP, (mmHg)^*^	131 (30)	136 (28)	131 (29)	122 (34)	0.06
Base excess (mEq/L)	−1.4 [−4.0–0.6]	−1.3 [−3.2–0.2]	−1.2 [−3.7–0.8]	−4.3 [−9.5—0.5]	<0.001
RBC (units)	5 [3–8]	4 [3–5]	4 [3–8]	6 [4–11]	<0.05
FFP (units)	4 [4–8]	3 [2–4]	4 [4–8]	6 [4–13]	0.001
PLT (units)	1 [1–2]	1 [1–1]	1 [1–2]	2 [1–5]	<0.01
Cryoprecipitate	2 [2–2]	NA	2 [2–2]	2.5 [2–5]	0.06

All variables expressed as median and interquartile ranges [IQR]. ^*^Expressed as mean and standard deviation (SD). ^1^Hypercoagulable *G* ≥11.7 dynes/cm^2^; ^2^normocoagulable *G* = 5–11.7 dynes/cm^2^; ^3^hypocoagulable *G* <5 dynes/cm^2^. ED: emergency department; ISS: injury severity score; BP: blood pressure; RBC: red blood cell; FFP: fresh frozen plasma; PLT: platelets; NA: not applicable.

### ROTEM profiles and hypercoagulability on admission

Baseline thromboelastometry data were available for 886 patients upon ED admission. On admission, the *G* value was increased in 63 (7%) of the patients, while 71 (8%) were hypocoagulable and the remaining 85% had normal clot strength according to the *G* value. Patients showing hypercoagulability on admission were more often female (40% vs. 28%, *P* <0.001), had lower ISS scores (9 vs. 20, *P* <0.001) and higher base excess values (−1.3 mEq/L vs. −4.3 mEq/L, *P* <0.001) compared to hypocoagulable patients. Also, they received less RBC, FFP and platelet transfusions compared to hypocoagulable patients. In addition, hypocoagulable patients had longer time to arrival at ED and a trend toward a higher incidence of TBI (Table 1).

### ROTEM profiles and hypercoagulability 24 hours after admission

Twenty-four hours after admission, for 451 out of 776 admitted patients, ROTEM profiles were available, 26 (6%) patients were hypercoagulable and 35 (8%) were hypocoagulable (Figure S1 in Additional file 1). In accordance with the hypercoagulable patients at ED admission, the hypercoagulable patients 24 hours after admission had lower ISS scores (14 vs. 25, *P* = 0.04), higher base excess values (−1.4 mEq/L vs. −6.2 mEq/L, *P* <0.001) and received less RBC transfusions compared to the hypocoagulable patients. Amount of FFP and platelets transfused did not differ between hyper-, normo- and hypocoagulable patients.

Altogether, during the first 24 hours after trauma, 88 (10%) patients were hypercoagulable at some point. Patients showing hypercoagulable ROTEM profiles had higher platelet counts and fibrinogen levels (Table S2 in Additional file 1).

### ROTEM profiles and multiple organ failure

**Figure 2:**
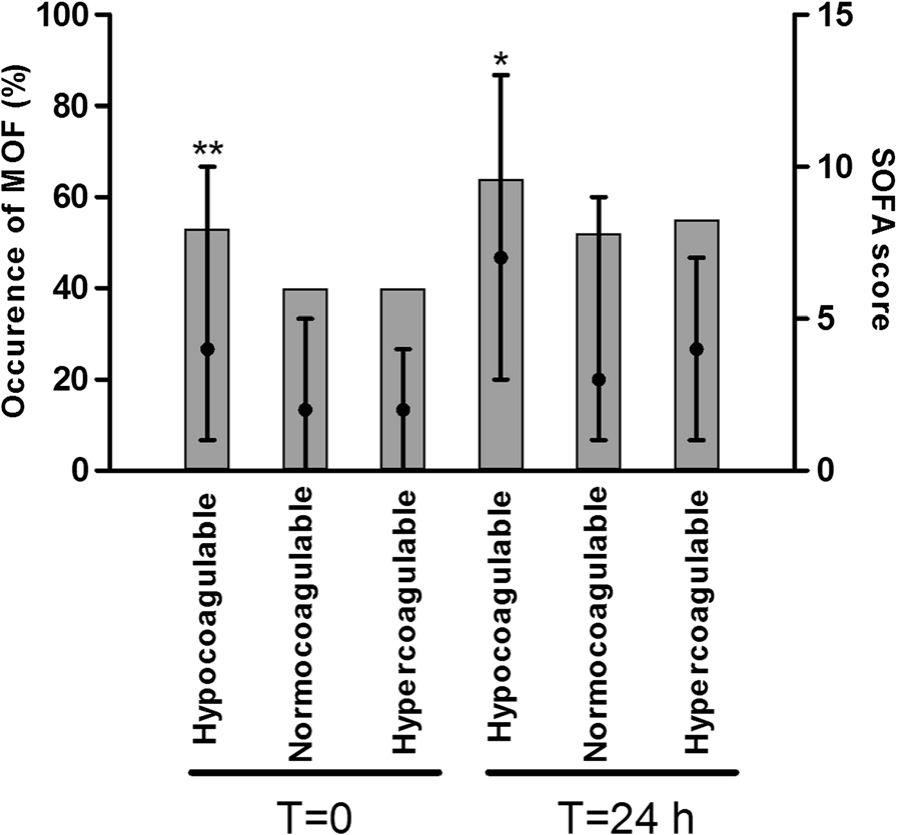
**Occurrence of multiple organ failure and the worst SOFA scores in patients with hypo-, normo- and hypercoagulable profiles at admission and 24 hours after admission.** Gray bars indicate occurrence of multiple organ failure and black dots indicate median SOFA scores and interquartile ranges. ^*^ *P* <0.01. ^**^ *P* <0.05. MOF: multiple organ failure; SOFA: sequential organ failure assessment.

Forty-one percent of trauma patients developed MOF (Figure 1). These patients were older, had higher ISS scores, more often had brain injury and received more blood products (Table S1 in Additional file 1). Of patients who were hyper- or normocoagulable on admission, 40% developed MOF, compared to 53% of the hypocoagulable patients. In patients presenting with hypocoagulability, the worst SOFA scores were higher compared to those who were normo- or hypercoagulable on admission (*P* = 0.003, Figure 2). Also, patients who developed MOF had hypocoagulable admission profiles as measured by ROTEM compared to patients who did not develop MOF (Table 2).

**Table 2:** **Thromboelastometry results at admission of patients who did and did not develop multiple organ failure**

	**MOF**	**No MOF**	***P*** **value**
	**N = 381**	**N = 549**	
INTEM CT (sec)	138 [115 to 168]	134 [113 to 166]	0.22
INTEM CFT (sec)	80 [63 to 104]	71 [60 to 89]	<0.001
INTEM alpha°	74 [70 to 77]	76 [73 to 78]	<0.001
INTEM MCF (mm)	60 [56 to 64]	62 [58 to 65]	<0.001
EXTEM CT (sec)	59 [49 to 73]	55 [46 to 68]	0.002
EXTEM CFT (sec)	98 [78 to 122]	88 [72 to 105]	<0.001
EXTEM alpha°	71 [66 to 75]	73 [69 to 76]	<0.001
EXTEM MCF (mm)	60 [56 to 65]	62 [58 to 66]	0.005
EXTEM *G* (dynes/cm^2^)	7.5 [6.4 to 9.3]	8.2 [6.9 to 9.7]	0.005

Median and interquartile range [IQR]. MOF: multiple organ failure; CT: clotting time; CFT: clot formation time; MCF: maximum clot firmness.

The same picture was noted 24 hours after admission. The worst median SOFA scores were highest among patients showing hypocoagulability 24 hours after admission, indicating more severe organ failure in these patients (Figure 2).

Conventional coagulation screens also indicated more hypocoagulable profiles in patients developing MOF (Table S3 in Additional file 1).

### ROTEM profiles and prediction of MOF and mortality

Univariate logistic regression analysis with admission ROTEM variables identified INTEM CFT, INTEM alpha, INTEM MCF, EXTEM CT, EXTEM alpha, EXTEM MCF and *G* to be associated with the occurrence of MOF, as were trauma characteristics and baseline vital parameters. After performing multiple logistic regression analysis with ROTEM variables, admission EXTEM CT, alpha and *G* were shown to be predictors for the occurrence of MOF (Table 3). The odds ratios for MOF indicated that change of the parameters toward a more hypocoagulable profile resulted in an increased risk for the development of MOF. We did not find any correlation between a hypercoagulable profile and the occurrence of MOF.

**Table 3:** **Prediction of occurrence of multiple organ failure by EXTEM ROTEM variables at admission and 24 hours after admission with multivariate analysis**

	**OR**	**95% CI**	***P*** **value**
**Admission**			
CT	1.01	1.00-1.01	0.05
CFT	0.99	0.99-1.00	0.23
Alpha	0.95	0.92-0.98	<0.01
MCF	1.01	0.98-1.04	0.62
*G*	0.94	0.89-0.99	0.02
**24 hours after admission**			
CT	1.00	0.00-2.39	0.86
CFT	1.03	1.01-1.04	<0.01
Alpha	1.04	0.95-1.15	0.37
MCF	1.04	0.99-1.09	0.13
*G*	0.92	0.85-1.00	0.05

OR: odds ratio; CI: confidence interval; CT: clotting time; CFT: clot formation time; MCF: maximum clot firmness.

EXTEM CFT and *G* were predictors for MOF 24 hours after admission (Table 3).

The total mortality was 10% (n = 97) (Figure 1). Of note, patients who were hypercoagulable on admission had lower 28-day mortality compared to normo- and hypocoagulable patients (0% in hypercoagulable patients vs. 7% in normocoagulable and 24% in hypocoagulable patients, *P* <0.001). Multivariate analysis with ROTEM variables showed that low EXTEM alpha angle on admission was a predictor for mortality (0.95 (0.91 to 0.98) *P* <0.01). Every degree increase of the alpha angle results in a 0.95 reduction of mortality risk.

### ROTEM profiles, transfusion strategy and occurrence of MOF

In order to assess whether liberal use of FFP affected occurrence of hypercoagulability and subsequent MOF, we performed an additional subanalysis in patients transfused with RBC and FFP. Transfused patients were divided in one group with an RBC:FFP ratio of 1:1 (n = 35), one with a ratio of more than 1:1 (n = 115) and one with a ratio of less than 1:1 (n = 21). These three groups did not differ significantly with respect to baseline characteristics (data not shown) and platelet transfusions. ROTEM EXTEM CT, CFT, MCF and alpha did not differ at baseline and after 24 hours, nor did *G* values (data not shown). After 24 hours, none of the patients transfused with a RBC:FFP ratio <1:1 showed hypercoagulability and of patients transfused with higher ratios of RBC:FFP, only two out of one hundred patients progressed from a normocoagulable to a hypercoagulable state (Figure S2 in Additional file 1).

Occurrence of MOF was high in all groups, but did not differ between groups with different transfusion ratios (82% in patients with a ratio of 1:1 or higher, and 81% in patients with RBC:FFP <1:1 respectively, *P* = 0.99).

## Discussion

The current study shows that a hypercoagulable state as detected by thromboelastometry, occurred in 7% at admission and in 10% of patients within 24 hours after trauma. Characteristics associated with the presence of hypercoagulability included lower ISS, higher base excess values, female gender and shorter time to ED arrival. These rates were not that different to the detected incidence for hypocoagulability, which has been the focus of considerable research interest, as part of evolving concepts of ATC. In contrast to our hypothesis, hypercoagulability did not appear to predict the occurrence of MOF. Rather, severity of MOF after trauma was associated with a hypocoagulable state. Hypercoagulable patients at admission had lower mortality, consistent with lower ISS and more normal base excess values. High EXTEM CFT and low *G* values were predictive for the development of MOF and low EXTEM alpha was predictive for mortality.

Hypercoagulability after trauma has been reported using a variety of thrombelastographic (TEG) measurements [5,7,9,25,26], however, whether TEG and ROTEM results are interchangeable is still under debate [27,28]. Reported incidences of hypercoagulability diagnosed by TEG range from 11 to 80% [5,7,9,10,26]. This wide variation can be ascribed to use of different definitions of hypercoagulability, with studies using individual parameters of the thrombelastographic trace [5,9,26], a combination of parameters [6,10] or the use of *G* as a marker of whole clot strength [7]. Also injury severity and timing of measurements differed among these cohorts. Although our findings are in line with those previously reported in a smaller cohort of trauma patients [26], our observed rates of hypercoagulability were lower compared to a recent evaluation of admission profiles with TEG, most likely due to the use of a more narrow definition of hypercoagulability in this study [9]. With respect to the observed rate of hypocoagulability, our numbers were in line with the previous mentioned TEG study [9], but lower than other reports from the ACIT cohort using ROTEM [29], because only patients needing more than four units of RBC were included.

We hypothesized that occurrence of hypercoagulability was associated with MOF. However, we observed an opposite effect. Patients showing hypocoagulability within the first 24 hours of admission developed more severe organ failure and had an increased late mortality. This observation is in line with studies demonstrating that hypocoagulability is associated with adverse outcome after trauma and brain injury [23,30,31]. We showed that, in addition to individual parameters, *G* values on admission and 24 hours after admission are predictors for the occurrence of MOF. Of note, *G* is considered to better represent total clot strength than the individual thrombelastography parameters [7,16,23]. Previous studies on ROTEM in trauma patients focused on diagnosing early coagulation abnormalities [20,32], prediction of transfusion requirements [21,33] and correction of hypocoagulability [34,35]. Our data indicate, contrary to our hypothesis, that hypocoagulability detected by ROTEM also has an enhanced risk of adverse late outcome, which confirms previous observations in a smaller cohort [21].

In the current study, patients with hypercoagulability had lower mortality and lower SOFA scores. A similar observation was recently reported in a smaller cohort of trauma patients [9]. Therefore, we hypothesize that early hypercoagulability after trauma is an evolutionary response to prevent exsanguination. Results do not point toward the hypothesis that early hypercoagulability after trauma resembles disseminated intravascular coagulation (DIC) with the formation of microthrombi, thereby contributing to organ failure [15,36]. The observation that early hypercoagulability after trauma is more prevalent in females is in line with a previous report [5].

Limitations of our study include that we did not systematically look for occurrence of venous thromboembolism as this is a complication of major interest in patients initially surviving major trauma. In addition, only the summary of the SOFA score was collected, hereby we cannot comment on SOFA subgroups. It is also not possible to rule out a contribution of late hypercoagulability to the development of organ failure, as we only assessed ROTEM on admission and after 24 hours. Prolonged hypercoagulability has been linked to increased risk of thromboembolic complications [7,10,11]. Also, we did not assess d-dimers and hereby we were not able to correlate ROTEM findings to DIC scores. However, a recent review of pathology samples obtained early after trauma failed to demonstrate microthrombi despite the clinical presence of increased DIC scores [37]. Furthermore, in 40% of admitted patients ROTEM values were not available at 24 hours following trauma, attributed to logistic issues, which could have introduced recruitment bias and contributed to an underestimation of hypercoagulability at this time point. Limited reports have described the coagulopathic changes over time in trauma and a recent small cohort study suggested that hypercoagulability after trauma occurs after 48 hours [38]. Therefore, further research should include serial measurements and a prospective standardized observation of complications after trauma. However, the current data suggest that early hypercoagulability after trauma not only reduces early mortality [9], but also seems to be associated with lower occurrence and severity of MOF and 28-day mortality.

Altogether, this study has identified a significant proportion of patients with hypercoagulability as defined by ROTEM at admission. Further work in larger studies should define the clinical consequences and prognostic value of identifying hypercoagulability, specifically including thromboembolic events, and might assess a role for very early, targeted use of anticoagulants in selected patients. The role of plasma or other blood components in potentially exacerbating the consequences of hypercoagulability is also an area of further research. In this study, patients with hypocoagulability on admission mostly tended to regress to normal values over time and not to hypercoagulability, irrespective of blood product ratio. This is in contrast with studies showing an association between amount of blood products and MOF [39], but is in line with other studies, which suggested that other fluids were more associated with MOF than blood products [40,41]. Also, there is experimental evidence that FFP preserves endothelial integrity in hemorrhagic shock [42].

## Conclusions

In a cohort of trauma patients, 10% shows a hypercoagulable state, as defined by ROTEM *G* value, within the first 24 hours. Occurrence of early hypercoagulability is not associated with development of MOF, moreover it appears to protect against adverse outcome. Admission ROTEM variables indicating hypocoagulability are predictive of the development of MOF and mortality. Liberal use of FFP is not associated with enhanced hypercoagulability.

## Key messages

- Up to 10% of trauma patients show hypercoagulable ROTEM profiles within the first 24 hours after admission.
- Hypocoagulable, but not hypercoagulable profiles in trauma patients, are associated with adverse outcome.
- Further research on the prognostic value of ROTEM profiles in trauma patients is warranted and should focus on sequential measurements to further elucidate coagulation patterns and their influence on patient outcomes.

## Additional file

Additional file 1:
**Table S1.** Characteristics of patients who did and did not develop multiple organ failure. **Table S2.** Fibrinogen levels and platelet count in hypo-, normo- and hypercoagulable patients at admission and 24 hours after admission. **Table S3.** Conventional coagulation test results at admission in patients who did and did not develop multiple organ failure. **Figure S1.** ROTEM measurements in trauma patients at admission and 24 hours after admission. Profiles classified as hyper-, normo- or hypercoagulable according to *G* value. **Figure S2.** ROTEM measurements in trauma patients transfused with RBC and FFP at admission and 24 hours after admission. Profiles classified as hyper-, normo- or hypercoagulable according to *G* value.

## Abbreviations

ACIT: Activation of Coagulation and Inflammation in Trauma
ATC: acute traumatic coagulopathy
CFT: clot formation time
CT: coagulation time
DIC: disseminated intravascular coagulation
ED: emergency department
FFP: fresh frozen plasma
IQR: interquartile range
ISS: injury severity score
MCF: maximum clot firmness
MOF: multiple organ failure
PLT: platelets
RBC: red blood cells
ROTEM: thromboelastometry
SOFA: sequential organ failure assessment
TBI: traumatic brain injury
TEG: thromboelastography
