# Supplementary material for: Thromboelastometry and organ failure in trauma patients: a prospective cohort study
Source: Crit Care. 2014 Dec 25;18(6):687. doi: 10.1186/s13054-014-0687-6 (PMC4305250; doi:10.1186/s13054-014-0687-6)
Supplement: Additional file 1: — Table S1. Characteristics of patients who did and did not develop multiple organ failure. Table S2. Fibrinogen levels and platelet count in hypo-, normo- and hypercoagulable patients at admission and 24 hours after admission. Table S3. Conventional coagulation test results at admission in patients who did and did not develop multiple organ failure. Figure S1. ROTEM measurements in trauma patients at admission and 24 hours after admission. Profiles classified as hyper-, normo- or hypercoagulable according to G value. Figure S2. ROTEM measurements in trauma patients transfused with RBC and FFP at admission and 24 hours after admission. Profiles classified as hyper-, normo- or hypercoagulable according to G value. [file 13054_2014_687_MOESM1_ESM.docx]

**Additional file 1**

**Table S1** Characteristics of patients who did and did not develop multiple organ failure.

|  | **MOF**  **N = 381** | **No MOF**  **N = 549** | ***p* value** |
| --- | --- | --- | --- |
| Age (years) | 40 [27-57] | 35 [24-49] | <0.001 |
| Sex, male % (n) | 77 (303) | 80 (422) | 0.41 |
| Time to ED (minutes) | 78 [60-95] | 65 [47-83] | <0.001 |
| Trauma mechanism, blunt % (n) | 87 (327) | 79 (430) | 0.001 |
| Brain injury, % (n) | 42 (160) | 15 (80) | <0.001 |
| Injury severity score | 24 [13-34] | 9 [4-17] | < 0.001 |
| Systolic blood pressure (mmHg) | 124 (32) | 134 (27) | <0.001 |
| Base Excess (mEq/L) | -3 [-6.35 - -0.80] | -0.45 [-2.43 - 1.13] | <0.001 |
| Red Blood Cells |  |  |  |
| Patients transfused, % (n) | 44 (168) | 12 (65) | <0.001 |
| Units | 6 [4-10] | 3 [2-6] | <0.001 |
| Fresh frozen plasma |  |  |  |
| Patients transfused, % (n) | 34 (130) | 6 (30) | <0.0001 |
| Units | 5 [4-8] | 4 [2-4] | <0.001 |
| Platelets |  |  |  |
| Patients transfused, % (n) | 24 (92) | 3 (16) | <0.0001 |
| Units | 1 [1-2] | 1 [1- 1] | 0.05 |

Data expressed as median [interquartile ranges] or mean (standard deviation).

ED = emergency department.

MOF = multiple organ failure

**Table S2** Fibrinogen levels and platelet count in hypo-, normo- and hypercoagulable patients at admission and 24 hours after admission.

|  | **Hypocoagulable** | **Normocoagulable** | **Hypercoagulable** |
| --- | --- | --- | --- |
| **Admission** | **N=71** | **N=752** | **N=63** |
| Fibrinogen (g/L) | 1.8 [1–2.3] | 2.3 [1.9–2.8] | 3.2 [2.6–3.8] |
| Platelet count (*10^9^/L) | 188 [136–244] | 225 [190–266] | 275 [249–344) |
| **24 h after admission** | **N=35** | **N=390** | **N=26** |
| Fibrinogen (g/L) | 1.9 [1.7–2.4] | 3.3 [2.7–3.8] | 3.8 [3.1–4.0] |
| Platelet count (*10^9^/L) | 87 [63–124] | 162 [122–198] | 226 [202–246] |

Data expressed as median and interquartile ranges.

**Table S3** Conventional coagulation test results at admission in patients who did and did not develop multiple organ failure.

|  | **Multiple Organ Failure**  **N=381** | **No Multiple Organ Failure**  **N=549** | ***p* value** |
| --- | --- | --- | --- |
| INR | 1.1 [1-1.2] | 1.0 [1-1.1] | <0.001 |
| PT (sec) | 11.4 [10.9-12.4] | 11.1 [10.6-11.7] | <0.001 |
| Platelet count (*10^9^/L) | 219 [176-256] | 239 [196-277] | <0.001 |
| Fibrinogen (g/L) | 2.0 [1.6-2.6] | 2.5 [2.1-2.9] | <0.001 |

Data expressed as median and interquartile ranges.

**Figure S1** ROTEM measurements in trauma patients at admission and 24 hours after admission. Profiles classified as hyper-, normo- or hypercoagulable according to *G* value.


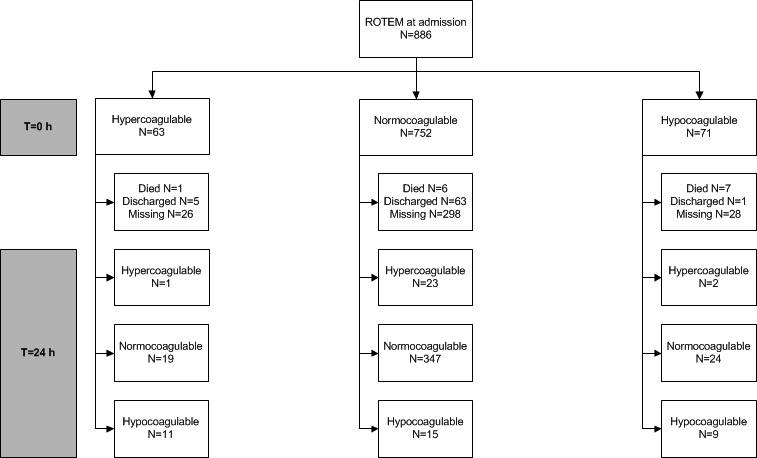


Hypercoagulable *G*>11.7 dynes/cm^2^ ; normocoagulable *G*=5-11.7 dynes/cm^2^ ; hypocoagulable *G*<5 dynes/cm^2^

**Figure S2** ROTEM measurements in trauma patients transfused with RBC and FFP at admission and 24 hours after admission. Profiles classified as hyper-, normo- or hypercoagulable according to *G* value.


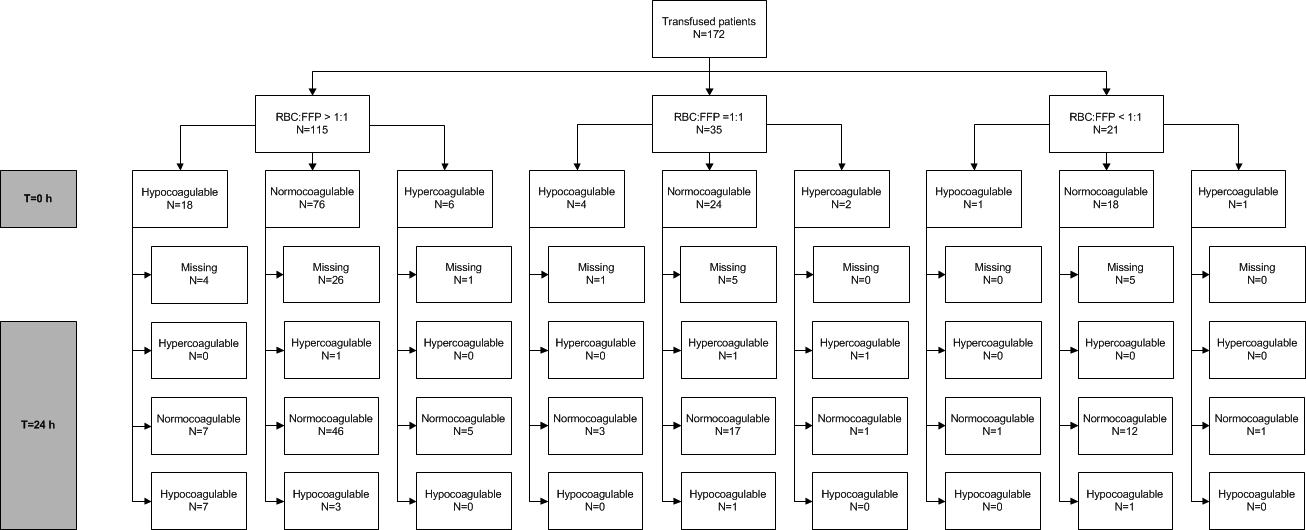


Hypercoagulable *G*>11.7 dynes/cm^2^; normocoagulable *G*=5-11.7 dynes/cm^2^ ; hypocoagulable *G*<5 dynes/cm^2^
